# Behavioral signatures related to genetic disorders in autism

**DOI:** 10.1186/2040-2392-5-11

**Published:** 2014-02-11

**Authors:** Hilgo Bruining, Marinus JC Eijkemans, Martien JH Kas, Sarah R Curran, Jacob AS Vorstman, Patrick F Bolton

**Affiliations:** 1 Brain Center Rudolf Magnus, Department of Psychiatry, University Medical Center, Postbus 85500, Heidelberglaan 100 3508 GA, Utrecht, The Netherlands; 2 Brain Center Rudolf Magnus, Department of Translational Neuroscience, Utrecht, The Netherlands; 3 Julius Center for Health Sciences and Primary Care, University Medical Center, Utrecht, The Netherlands; 4 King’s College London, Institute of Psychiatry, De Crespigny Park, London, UK

## Abstract

**Background:**

Autism spectrum disorder (ASD) is well recognized to be genetically heterogeneous. It is assumed that the genetic risk factors give rise to a broad spectrum of indistinguishable behavioral presentations.

**Methods:**

We tested this assumption by analyzing the Autism Diagnostic Interview-Revised (ADI-R) symptom profiles in samples comprising six genetic disorders that carry an increased risk for ASD (22q11.2 deletion, Down’s syndrome, Prader-Willi, supernumerary marker chromosome 15, tuberous sclerosis complex and Klinefelter syndrome; total n = 322 cases, groups ranging in sample sizes from 21 to 90 cases). We mined the data to test the existence and specificity of ADI-R profiles using a multiclass extension of support vector machine (SVM) learning. We subsequently applied the SVM genetic disorder algorithm on idiopathic ASD profiles from the Autism Genetics Resource Exchange (AGRE).

**Results:**

Genetic disorders were associated with behavioral specificity, indicated by the accuracy and certainty of SVM predictions; one-by-one genetic disorder stratifications were highly accurate leading to 63% accuracy of correct genotype prediction when all six genetic disorder groups were analyzed simultaneously. Application of the SVM algorithm to AGRE cases indicated that the algorithm could detect similarity of genetic behavioral signatures in idiopathic ASD subjects. Also, affected sib pairs in the AGRE were behaviorally more similar when they had been allocated to the same genetic disorder group.

**Conclusions:**

Our findings provide evidence for genotype-phenotype correlations in relation to autistic symptomatology. SVM algorithms may be used to stratify idiopathic cases of ASD according to behavioral signature patterns associated with genetic disorders. Together, the results suggest a new approach for disentangling the heterogeneity of ASD.

## Background

Autism spectrum disorder (ASD) is a behaviorally defined syndrome characterized by variable abnormalities in social interactions and communication, in association with restricted interest patterns and unusual stereotyped behaviors. There has been a concerted effort over the last 20 years to identify causal genetic risk factors and as a result, an increasing number of rare, highly penetrant genetic variants are being implicated [1]. When present, these rare variants are thought to account for a large proportion of an individual’s genetic liability to the condition. Currently, specific genetic etiologies, including rare single nucleotide and copy number variants (CNVs) as well as larger chromosomal variations, can be identified in around 15 to 20% of patients [2-5]. These findings highlight the complexity of the genetic architecture and heterogeneity of ASD and indicate that by using standard case–control designs, extremely large sample sizes will be required to unravel the heterogeneity and map the dysregulated signaling pathways involved in the pathophysiology of ASD [4,6-9].

The variability in phenotypic expression of autism observed in monozygotic twin pairs, coupled with the evidence from molecular genetic studies supporting a polygenic multi-factorial liability model has led to the recognition that the many genetic risk factors for autism give rise to a broad spectrum of behavioral presentations and hence the concept of autism as a spectrum disorder. The adoption of this model has led to an implicit assumption that specific genotype-phenotype correlations are unlikely to exist. However, there is evidence that ASD symptoms may be dissociable at the genetic level. Different genetic linkage regions have been obtained for social interaction and repetitive behavioral domains in ASD patients [10], and distinct developmental trajectories of social and repetitive behavior exist in the ASD population [11]. Moreover, in recent years, a growing interest has developed in the possibility that particular genetic disorders may give rise to characteristic patterns of autistic symptomatology. This interest is based on the assumption that perturbations in associated pathophysiological pathways would lead to relatively constrained and more specific phenotypic outcomes [12]. Indeed, a number of recent studies, involving a variety of genetic conditions including 16p11.2 and 7q11.23 CNVs, Williams syndrome, fragile X syndrome and neurofibromatosis, have indicated the existence of genetic disorder-specific behavioral profiles that encourage further efforts in this direction [4,13-16]. Building on these findings, we postulated that well-defined genetic conditions could give rise to relatively distinct patterns of autistic symptomatology. The designation of these patterns may be relevant to dissect ASD heterogeneity as other risk factors that perturb converging pathophysiological pathways, for example related to the genetic conditions, might lead to similar patterns of autistic symptomatology.

**Figure 1:**
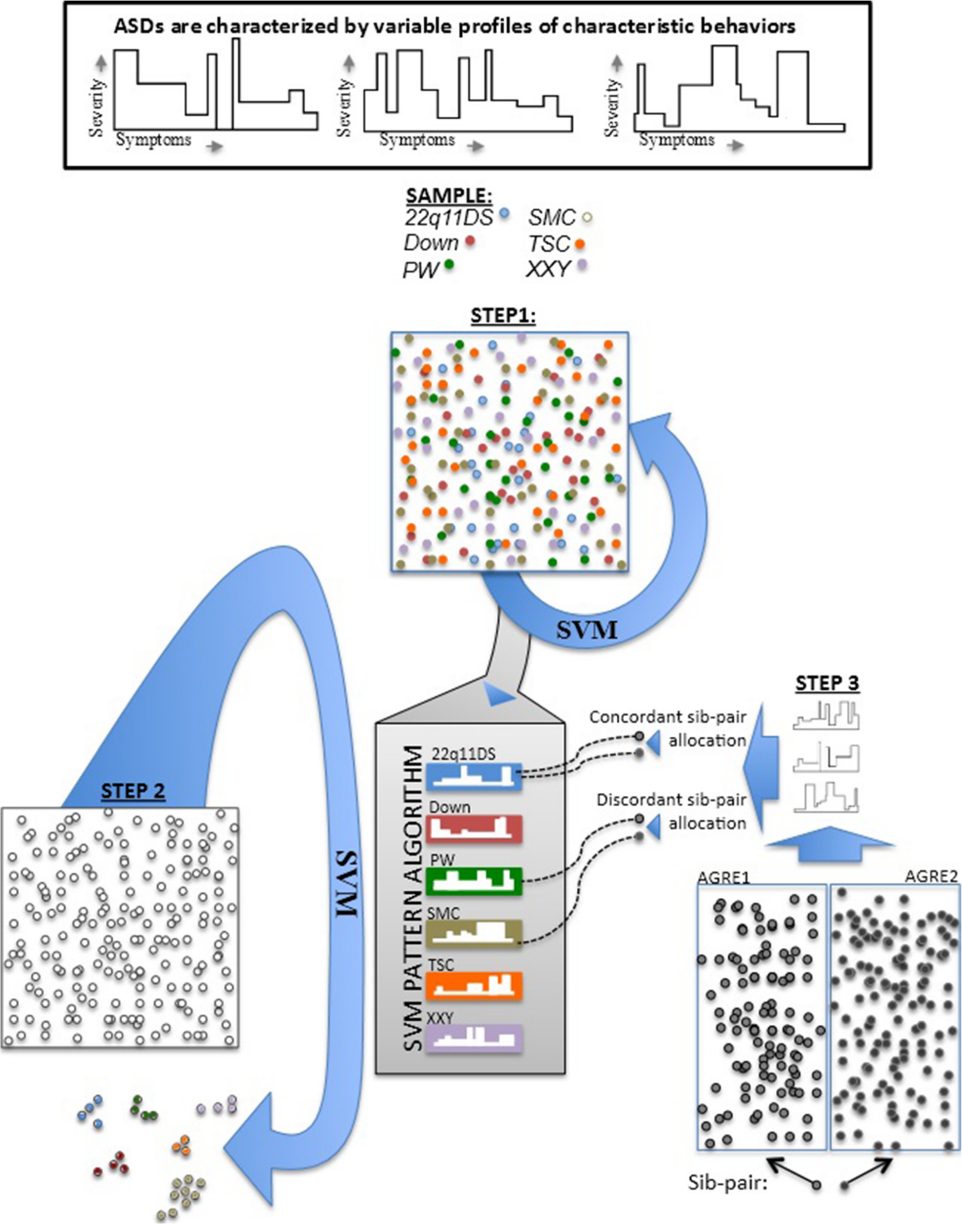
**Overview of the different steps undertaken in the study.** Step 1: development of SVM classifier to assess the presence and strength of behavioral signatures among genetic syndromes. Step 2: application of the classifier derived in step 1 to AGRE samples to test if similarity in behavioral signatures can be detected among idiopathic ASD subjects. Step 3: application of classifier derived in step 1 to sibling pairs with idiopathic ASD (AGRE) to test relative familiality of behavioral signatures derived from genetic syndromes. AGRE, Autism Genetics Resource Exchange; ASD, autism spectrum disorder; SVM, support vector machine.

In the present study, we have undertaken a proof of concept study to determine if these genotype-phenotype correlations exist and whether they could be useful to disentangle the heterogeneity of ASD and complement future genetic studies. Support vector machine (SVM) learning was used to analyze ‘signatures’ of autistic symptomatology in six genetic developmental disorders associated with an increased risk for ASD [17-20]. Based on the premise that other risk factors which dysregulate the same pathways may give rise to similar ‘signature’ patterns of behavior, we aimed to apply the SVM algorithms derived from genetic disorders to cases of idiopathic ASD. Finally, we investigated whether the SVM algorithm would detect enhanced behavioral similarity in affected sib pairs from the Autism Genetics Resource Exchange (AGRE) multiplex families. Figure 1 provides an overview of the different steps involved in the study.

## Methods

### Subjects

The six genetic disorders we included in the study were: 22q11.2 deletion syndrome (22q11DS), Down’s syndrome (DS) [21], Prader-Willi syndrome (PWS), supernumerary marker chromosome 15 (SMC15), tuberous sclerosis complex (TSC) and Klinefelter syndrome (XXY); total n = 322 cases, groups ranging in sample size from 21 to 90 cases. Cases were recruited through patient associations/charities or centers for clinical genetics or pediatrics as part of a collaborative effort between the Department of Psychiatry of the University Medical Centre in Utrecht in the Netherlands and the Institute of Psychiatry, King’s College London in the UK. Appropriate local ethical board approval was obtained (Medical Research Ethics Committee, METC, of the University Medical Centre in Utrecht and the College Research Ethics Committee, CREC, in London). Informed consent for each participant in the cohorts was obtained and included the use of data for the analysis we carried out for this paper. The genetic disorders had been diagnosed through clinical genetic centers and confirmed by routine molecular and cytogenetic analysis. The total sample consisted of 322 verbal subjects. Each of the six genetic disorders has previously been shown to be associated with an increased risk of ASD [6,7,22-25]. The cases were drawn from studies that had originally been designed to elucidate the behavioral phenotypes associated with each of the six genetic disorders [22-27]. As far as possible, the samples were ascertained without reference to the presence of ASD. For more details on recruitment procedures and inclusion criteria for the genetic disorder subtypes please see previous publications [22-26]. All subjects were included in the analyses, regardless of the presence of an ASD diagnosis, in order to evaluate the widest range of symptom profiles. However, for technical reasons concerning the measurement of ASD symptomatology, only verbal individuals were included in the analyses. Estimates of intellectual abilities were available for the majority of subjects (>80%) and had been assessed by different standardized measures according to age and ability level [28-32]. Table 1 shows the sample characteristics.

**Table 1:** Characteristics of the total genetic disorder sample

**Genetic disorder**	**N**			**Age (months)**	**ASD**		**ADI-R scores per domain and total scores**				**IQ**
	**Total**	**Female**	**Male**		**Yes**	**No**	**I**	**II**	**III**	**Total**	
22q11DS	90	42	48	162.5 ± 33.6	40	50	9.8 ± 6.4	7.7 ± 4.8	2.5 ± 2.2	20.0 ± 12.0	67.0 ± 14.1
Down’s	21	16	5	169.1 ± 32.6	6	15	7.2 ± 4.4	6.8 ± 3.8	3.2 ± 2.0	17.2 ± 8.6	49.5 ± 11.9
PWS	88	48	40	191.9 ± 141.0	20	68	7.9 ± 5.1	5.7 ± 4.5	2.8 ± 2.0	16.3 ± 10.1	70.9 ± 16.3
SMC15	22	8	14	161.4 ± 103.6	19	3	15.6 ± 6.0	13.6 ± 5.5	6.5 ± 2.4	35.7 ± 12.2	51.0 ± 19.0
TSC	50	31	19	126.2 ± 74.0	22	28	12.0 ± 9.0	9.6 ± 6.8	3.7 ± 3.3	25.2 ± 17.9	69.3 ± 27.4
XXY	51	0	51	145.4 ± 41.4	16	35	8.5 ± 6.0	8.8 ± 5.4	2.3 ± 2.1	19.6 ± 12.0	80.4 ± 13.9
Total	322	145	177		123	199					
Average				162.7 ± 89.8			9.6 ± 6.7	8.0 ± 5.6	3.1 ± 2.5	20.6 ± 13.4	68.6 ± 19.2

Data provided are mean values and, if applicable, standard error of the means. ADI-R domains: I, qualitative abnormalities in reciprocal social interaction; II, qualitative abnormalities in communication; and III, restricted, repetitive and stereotyped patterns of behavior. 22q11DS, 22q11.2 deletion syndrome; ADI-R, Autism Diagnostic Interview-Revised; ASD, autism spectrum disorder; Down’s, Down’s syndrome; PWS, Prader-Willi syndrome; SMC15, supernumerary marker chromosome 15; TSC, tuberous sclerosis complex; XXY, Klinefelter syndrome.

The AGRE database was used for the selection of idiopathic subjects (http://www.agre.org) [33,34]. AGRE cases were included in the analyses if they fulfilled Autism Diagnostic Interview-Revised (ADI-R) criteria for an ASD and complete ADI-R algorithm data were available (see criteria). All verbal simplex probands in the AGRE cohort with complete ADI-R algorithm data and scoring above the ASD threshold (n = 375) were assigned the label ‘AGRE0’. Among the multiplex families we identified all verbal affected sib pairs. Within these affected pairs one sib was allocated to ‘AGRE1’ while the other was allocated to ‘AGRE2’. Therefore, AGRE1 and AGRE2 consisted of those verbal subjects with ASD with at least one related verbal sibling with ASD (both n = 433).

### Measures

Autism symptom variables were extracted from the ADI-R which was used to interview the parents of each subject [35]. The ADI-R is an established interview schedule for assessing autism diagnoses but may also be used to assess profiles of autistic symptomatology [36,37], and as phenotype variables in large genetic population studies of ASD [38-41]. The interview focuses on identifying key symptoms that characterize the syndrome [12,36,37]. A subset of 37 items from the ADI-R is used to create a diagnostic algorithm, which documents behaviors reported between the 4th and 5th birthday, regarded as the optimal window to detect ASD. As a consequence, the use of the diagnostic algorithm data minimalizes the possible confound of age-related developmental effects on symptomatology. ADI-R items are scored as: 0, no ASD behavioral symptom present; 1, specified behavior definitely present but not clearly enough to warrant a code of 2; or 2, specified ASD symptom definitely present. In addition, for some items a code of 3 is given, if the behavior impacts markedly on or disrupts family life. Accordingly, when computing the algorithm scores, a code 3 is recoded as a 2. For this study, we used these algorithm scores, with a range of 0 to 2 instead of 0 to 3, to assign equal weight to all items entered in the analyses. Because certain symptoms of the communication impairments characterizing ASD can only be observed in verbal individuals, there are separate scores for verbal and non-verbal individuals. An overview of the description of the ADI-R items and the ADI-R domains of the algorithm is provided in Table 2. The classification of an ASD in this study was based on ADI-R criteria used in genetic studies and the AGRE collection: ASD is diagnosed when scores in all domains are met or when scores are met in two core symptom domains, in addition to the ‘age of onset’ domain, but are one point away from meeting autism criteria in the one remaining core symptom domain [35,42]. Reliability of the ADI-R in a population with mild to moderate mental retardation has been established [43].

**Table 2:** Autism Diagnostic Interview-Revised (ADI-R) algorithm items sorted by number

**Item number**	**Item description**	**ADI-R domain**
31	Use of other’s body to communicate	I
33	Stereotyped utterances and delayed echolalia	III
34	Social verbalization/chat	II
35	Reciprocal conversation	II
36	Inappropriate questions or statements	II
37	Pronominal reversal	II
38	Neologisms/idiosyncratic language	II
39	Verbal rituals	III
42	Pointing to express interest	II
43	Nodding	II
44	Head shaking	II
45	Conventional/instrumental gestures	II
47	Spontaneous imitation of actions	II
48	Imaginative play	II
49	Imaginative play with peers	I
50	Direct gaze	I
51	Social smiling	I
52	Showing and directing attention	I
53	Offering to share	I
54	Seeking to share enjoyment with others	I
55	Offering comfort	I
56	Quality of social overtures	I
57	Range of facial expressions used to communicate	I
58	Inappropriate facial expressions	I
59	Appropriateness of social responses	I
61	Imitative social play	II
62	Interest in children	I
63	Response to approaches of other children	I
64	Group play with peers (age <10.0 years)	I
65	Friendships (age >10.0 years)	I
67	Unusual preoccupations	III
68	Circumscribed interests	III
69	Repetitive use of objects or interest in parts of objects	III
70	Compulsions/rituals	III
71	Unusual sensory interests (highest score of 69/71)	III
77	Hand and finger mannerisms (highest score of 77/78)	III
78	Other complex mannerisms or stereotyped body movements	III

ADI-R, Autism Diagnostic Interview-Revised.

### Statistical analysis

Standard principal component analysis (PCA) of ADI-R item scores was used to investigate the extent of overlap between the symptom profiles of the different genetic groups.

The SVM method was used as a supervised learning method (incorporating the knowledge of the genotype) to classify genotype membership on the basis of ADI-R item scores. SVM is currently one of the most popular machine learning methods used in data mining, due to its firm theoretical foundation and proven superiority in applications. With regards to SVM, a radial basis kernel function was used, with optimal gamma and cost parameter values determined in a nested n-fold or, equivalently, leave-one-out cross-validation (LOOCV) procedure, n being the number of observations in the sample. Each observation in turn was left out of the sample, and an SVM classifier was optimized and built on the remaining n - 1 observations. In this way, an independent assessment of correctness of the predicted class can be achieved for each observation in the sample, resulting in an independent estimate of the accuracy of SVM on the whole sample. In each one of the remaining samples, the optimization with respect to the gamma and cost parameter was achieved by applying a second LOOCV procedure, in which each of these n - 1 observations in turn was left out of the sample and SVM models were fitted to the remaining n - 2 observations, using a grid of combinations of gamma and cost parameter values. In a similar fashion as described above, accuracy was determined for every combination of gamma and cost parameter values on the grid, and the optimal value of gamma and cost parameter was determined as the one giving the highest accuracy. Finally, an SVM model was fitted to the n - 1 observations remaining in the outer loop using these optimal values. SVM by nature is a method for binary (two group) classification, so a multiclass (k classes) extension was used, based on the ‘one-against-one’ approach, in which k(k - 1)/2 binary classifiers are trained; the appropriate ‘predicted’ class is found by a voting scheme, choosing the most frequently assigned class by the binary classifiers.

Thus, the class assigned by SVM is the one with the maximum votes from all one-versus-one (2-group) classifications, based on the decision values of the 2-group classifiers. These decision values can also, *post hoc*, be used to obtain a predicted probability for each class, which can be used as outcome parameters to evaluate the confidence of SVM predictions.

The software used was the libSVM program, implemented through the SVM function in the e1071 library in R [44].

## Results

### Identification of behavioral signatures relating to each genetic disorder

**Figure 2:**
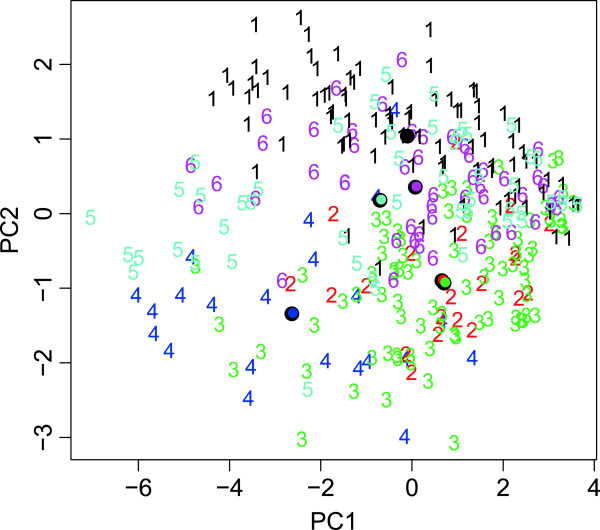
**PCA plot of ADI-R profiles of subjects in the genetic disorder sample.** Colors/numbers denoting genetic disorder subgroups. 1, 22q11.2 deletion syndrome; 2, Down’s syndrome; 3, Prader-Willi syndrome; 4, supernumerary marker chromosome 15; 5, tuberous sclerosis complex; 6, Klinefelter syndrome. ADI-R, Autism Diagnostic Interview-Revised; PCA, principal component analysis.

As a starting point, we explored the distribution of autism symptom profiles in the genetic disorder sample by PCA. The PCA plot showed that, on average, some genetic disorder profiles were overlapping where others were more clearly separable (Figure 2). This picture indicated that unsupervised statistical analysis was not sufficiently sensitive to optimally distinguish genetic disorder-related profiles. This notion was confirmed following cluster analysis (k-means clustering) of the ADI-R data in the genetic disorder sample, which did not identify any relevant clusters (data not shown).

To perform a more sophisticated pattern analysis, we turned to machine learning analysis. We used SVM as a supervised learning method to investigate genotype-phenotype relationships between the six genetic disorders and the item scores from the ADI-R algorithm. The essential difference with the unsupervised PCA or clustering analysis used above is that the SVM approach incorporates the knowledge of the genotype in the analysis. The SVM allocations to genetic disorder groups occurred in two steps. First, the SVM analyzed 2-group, ‘one-against-one’ comparisons. Subsequently, the multiclass extension was used to select the most appropriate ‘predicted’ genetic disorder class for each subject on the basis of the most frequently assigned class by the binary classifiers. The binary one-by-one comparisons showed high accuracies of up to 97% of correct genetic group allocations (Table 3). As a result, a total of 63% of cases was correctly allocated by the multiclass comparison using the LOOCV method, whereas random prediction (without prior knowledge of genetic group) would have resulted in 21% accuracy (Table 4). Interestingly, in all groups apart from DS, the averages of the post-hoc predicted probabilities were highest for the corresponding genetic disorder class, indicating that the SVM algorithm was able to predict correct disorder classes with a high degree of confidence (Table 4).

**Table 3:** One-by-one SVM comparisons in the genetic disorder sample

**Genotype**	**SVM accuracy (%)**					
	**22q11DS**	**Down’s**	**PWS**	**SMC15**	**TSC**	**XXY**
22q11DS	NA	0.89	0.91	0.97	0.90	0.82
Down’s	0.89	NA	0.77	0.84	0.82	0.87
PWS	0.91	0.77	NA	0.84	0.86	0.86
SMC15	0.97	0.84	0.84	NA	0.94	0.88
TSC	0.90	0.82	0.86	0.94	NA	0.72
XXY	0.82	0.87	0.86	0.88	0.72	NA

22q11DS, 22q11.2 deletion syndrome; Down’s, Down’s syndrome; NA, not applicable; PWS, Prader-Willi syndrome; SMC15, supernumerary marker chromosome 15; SVM, support vector machine; TSC, tuberous sclerosis complex; XXY, Klinefelter syndrome.

**Table 4:** Leave-one-out cross-validation (LOOCV) results for the SVM model on ADI-R items for the genetic disorder sample

**Genetic disorder**	**SVM frequency of assigned class and predicted probabilities**												
	**22q11DS**		**Down’s**		**PWS**		**SMC15**		**TSC**		**XXY**		**Total**
	n	Probability	n	Probability	n	Probability	n	Probability	n	Probability	n	Probability	n
22q11DS	**74**	**0.602**	1	0.092	7	0.095	0	0.032	6	0.15	14	0.215	102
Down’s	0	0.027	**1**	**0.166**	1	0.103	0	0.097	0	0.03	0	0.047	2
PWS	7	0.099	18	0.485	**68**	**0.537**	9	0.301	7	0.12	6	0.167	115
SMC15	0	0.02	0	0.11	4	0.089	**10**	**0.326**	0	0.05	2	0.067	16
TSC	2	0.103	0	0.044	3	0.068	0	0.064	**30**	**0.43**	9	0.185	44
XXY	7	0.149	1	0.103	5	0.108	3	0.179	7	0.22	**20**	**0.319**	43
Total	90		21		88		22		50		51		322
Accuracy	74/90 (82%)		1/21 (10%)		68/88 (77%)		10/22 (45%)		30/50 (60%)		20/51 (39%)		203/322 (63%)

22q11DS, 22q11.2 deletion syndrome; ADI-R, Autism Diagnostic Interview-Revised; Down’s, Down’s syndrome; LOOCV, leave-one-out cross-validation; PWS, Prader-Willi syndrome; SMC15, supernumerary marker chromosome 15; SVM, support vector machine; TSC, tuberous sclerosis complex; XXY, Klinefelter syndrome.

To further evaluate the validity of the prediction model, we investigated the correlation between the predicted probabilities and the proportion of cases correctly assigned to each genetic group, based on LOOCV output. This tests the expectation of the model that higher probabilities reflect greater confidence in prediction, as shown by increasing ‘correctness’ in classification. We observed a significant correlation (*P* = 0.002) between the predicted probabilities and the likelihood of correct classification, which provides support for the robustness of the model and encouraged us to test the classifier in further samples.

We were interested to identify which behaviors contributed most to the predictions by SVM. Therefore, the importance (weight) of each of the ADI-R items to the SVM classifier was extracted. The result of this analysis showed that four of the top five most influential items pertained to ASD symptoms that related to the quality of social interaction (Table 5). By contrast, the five least influential items were more concerned with aberrant communication and repetitive behaviors.

**Table 5:** ADI-R items that contributed most and least to the result of the SVM analysis on the genetic syndrome sample

**Lowest five ADI-R items**		**Top five ADI-R items**	
**Item number**	**Item description**	**Item number**	**Item description**
70	Compulsions/rituals	63	Response to approaches of other children
38	Neologisms/idiosyncratic language	49	Imaginative play with peers
58	Inappropriate facial expressions	64, 65	Group play with peers/friendships
39	Verbal rituals	56	Quality of social overtures
37	Pronominal reversal	68	Circumscribed interests

ADI-R, Autism Diagnostic Interview-Revised; SVM, support vector machine.

It was notable that the predicted probabilities in SMC15 cases were also relatively high for prediction to the PWS group. This seemed plausible, as both disorders are associated with differences in the ‘dosage’ of genes located in chromosome 15q11-13. By contrast, SMC15 could be clearly discriminated from 22q11DS by SVM, which corresponded with a lack of overlap in the PCA between these two groups (Figure 2). Interestingly, SMC15 and 22q11DS are both characterized by low average intelligence, suggesting that the behavioral differences are independent of general intellectual ability. To rule out the influence of IQ on prediction accuracy, we re-analyzed the data, including IQ as an additional predictor. The average accuracy of the SVM predictions was essentially unchanged (63.0% versus 62.5%), indicating that IQ was not a confounding factor. The poor prediction for the DS group was due to a frequent misallocation to the PWS group; 17 of the DS cases were being incorrectly assigned to the PWS group. Indeed, an overlap between DS and PWS groups was also apparent in the PCA of the symptom profiles (Figure 2).

We also tested the accuracy of SVM class assignment among the subset of individuals who scored above the ADI-R threshold for ASD (n = 123). This resulted in similar assignment accuracies and predicted probabilities (data not shown). In subsequent analyses we used the algorithm derived from all patients from our genetic disorder samples, irrespective of whether they met formal criteria for ASD diagnosis, since from a clinical perspective, we also wanted to include the profiles of subjects who scored below ADI-R thresholds for ASD.

### Testing the SVM classification algorithm in idiopathic ASD

**Figure 3:**
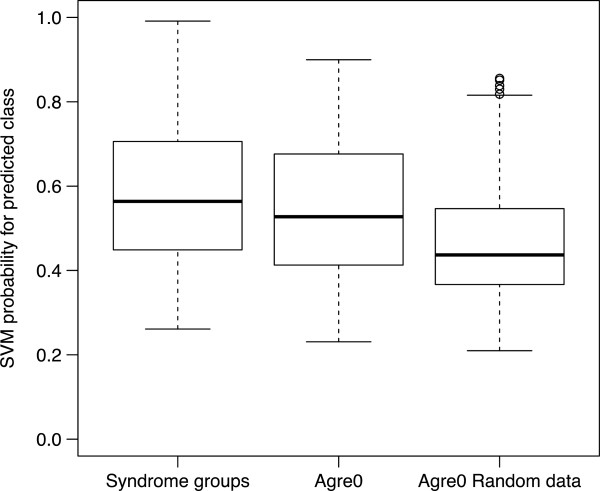
**SVM predicted probabilities of the original genetic groups, AGRE0 singleton dataset and randomly generated scores for the AGRE0 singleton dataset.** Mean SVM probabilities differed significantly between the genetic groups and AGRE0 (*P* = 0.0024), between the genetic groups and random data (*P* <0.001) and between AGRE0 and random data (*P* <0.001). SVM, support vector machine.

Next, we considered whether the genetic disorder algorithm could detect a degree of similarity in patterns of autistic behavior in a sample of ‘idiopathic’ cases. To test this hypothesis, we applied the algorithm to ADI-R data obtained from the AGRE dataset in order. It should be noted that the AGRE sample functioned as a ‘blind’ sample in this context, as we could not validate the outcome with genetic labels. Therefore, we performed analyses to indicate if the algorithm would detect meaningful associations or if these would not differ from random associations, for example not informed by genetic disorder labels. Thus, we generated randomly permuted ADI-R item data from the AGRE0 dataset and compared the distribution of predicted probabilities in the real (AGRE0 and genetic disorder sample) compared to the randomly generated data. The probabilities differed significantly between these groups. As expected, the highest predicted probabilities were observed among the genetic disorder cases. Indeed, the lowest probabilities were observed in the randomly generated AGRE subsample. There was also a significant difference between the genetic groups and AGRE0 (*P* = 0.0024), between the genetic groups and random data (*P* <0.001) and between AGRE0 and random data (Figure 3). Most importantly, the probabilities in AGRE0 were significantly higher than those in the randomly configured data (*P* <0.001). This indicated that the algorithm derived from the genetic disorders detected non-random pattern information.

**Figure 4:**
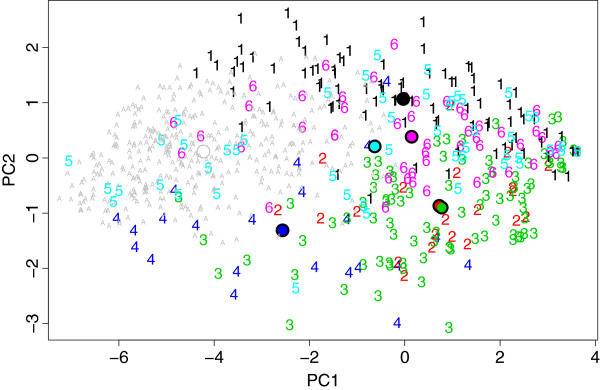
**PCA plot of ADI-R profiles of subjects in the genetic disorder sample, with the AGRE0 subsample inserted.** PC2 is the dimension with the most differentiating contrast among the genetic disorder groups. AGRE0, on average, has negative values on PC1 and is around 0 on PC2. The TSC group (5) is also on average 0 on PC2 similar to AGRE0 and has the most negative average on PC1. Groups 1, 4 and 6 also display some closeness to AGRE0. Colors/numbers/letters denote genetic disorder subgroups. 1, 22q11.2 deletion syndrome; 2, Down’s syndrome; 3, Prader-Willi syndrome; 4, supernumerary marker chromosome 15, 5, tuberous sclerosis complex, 6, Klinefelter syndrome; A, AGRE0. ADI-R, Autism Diagnostic Interview-Revised; PCA, principal component analysis; TSC, tuberous sclerosis complex.

Subsequently, we applied the genetic disorder classifier to the AGRE0 sample to analyze the distribution of genetic disorder allocations in the blind AGRE subsamples. The genetic disorder algorithm assigned the highest probabilities and most cases to the TSC group and the lowest probabilities and fewest cases to the DS and PWS groups. We observed a similar distribution of SVM predicted probabilities in the AGRE1 and AGRE2 samples, essentially replicating the result obtained for AGRE0. Again, TSC was by far the most commonly assigned class, whereas DS and PWS were the least frequently assigned classes. The predicted probabilities and group predictions for AGRE0, AGRE1 and AGRE2 are summarized in Table 6. It should be noted that these predictions were achieved by forcing all individuals into one of the six categories, which means that frequent allocation should be interpreted as indicative of relative phenotype similarity. As such, the application of the genetic disorder classifier to AGRE samples seemed to indicate enhanced relative similarity of AGRE profiles to the TSC group. To support this notion, we plotted the AGRE0 ADI-R profiles in the PCA plot of the genetic disorder sample, which confirmed that, on average, the TSC group displayed most similarity to AGRE0 (Figure 4). In addition, 22q11DS, SMC15 and XXY groups also displayed some closeness to AGRE0, which seems also reflected in their occasional allocation by the genetic disorder classifier.

**Table 6:** Application of the SVM algorithm derived from the genetic disorder sample to the different AGRE datasets

**Genetic disorder**	**AGRE0**				**AGRE1**				**AGRE2**			
	**n**	**% assigned**	**Mean probability**	**SD probability**	**n**	**% assigned**	**Mean probability**	**SD probability**	**n**	**% assigned**	**Mean probability**	**SD probability**
22q11DS	26	6.9	0.44	0.154	23	5.2	0.44	0.143	28	6.3	0.48	0.189
Down’s	1	0.3	0.25	NA	1	0.2	0.28	NA	1	0.2	0.27	NA
PWS	1	0.3	0.34	NA	5	1.1	0.30	0.131	5	1.1	0.33	0.072
SMC15	24	6.4	0.40	0.086	28	6.3	0.40	0.102	32	7.2	0.41	0.093
TSC	255	68	0.61	0.139	302	68.2	0.62	0.134	283	63.9	0.60	0.140
XXY	68	18.1	0.41	0.092	84	19	0.41	0.071	94	21.2	0.42	0.095
Total	375	100			443	100			443	100		

% assigned, percentage assigned to the respective genetic disorder class; 22q11DS, 22q11.2 deletion syndrome; AGRE, Autism Genetics Resource Exchange; Down’s, Down’s syndrome; mean probability, average predicted probability; n, number of assigned cases to the respective genetic disorder class; NA, not applicable; PWS, Prader-Willi syndrome; SD probability, standard deviation of predicted probability; SMC15, supernumerary marker chromosome 15; SVM, support vector machine; TSC, tuberous sclerosis complex; XXY, Klinefelter syndrome.

We contrasted these predictions in the AGRE sample with random predictions; we generated SVM models by randomly permuting the six labels relating to the genetic disorders. Thus, random genetic labels were linked to the existing symptom profiles, thereby destroying the original relationship between ADI-R score profiles and the genetic groups. By analyzing the allocations arising from these random classifier algorithms, we could check which distribution of allocation would arise by chance, that is not informed by existing genetic disorder profiles. We repeated this exercise 1,000 times in order to gain robust results. The results showed that most were assigned to the 22q11DS and PWS groups. This result was most likely due to the fact that these disorders were the two largest groups in the genetic disorder sample. It should be noted that this result was strikingly different than the allocation in AGRE by the randomly permuted genetic labels.

Together, these analyses on blind AGRE samples indicated that the algorithm of the genetic disorder sample could detect an extent of relative similarity in ADI-R profile patterns among idiopathic subjects.

### Behavioral signatures in sibling pairs with idiopathic ASD

To test our expectation that the signature patterns derived from the genetic disorders relate to genotype-phenotype associations, we hypothesized that the affected sib (sibling) would be significantly more often assigned to the same genetic disorder class and be relatively more similar in their behavioral profile than non-related subjects. To test this, we examined the concurrence in class assignment (X-square) and correlation between affected sib pairs in the SVM assigned class and predicted probabilities.

**Figure 5:**
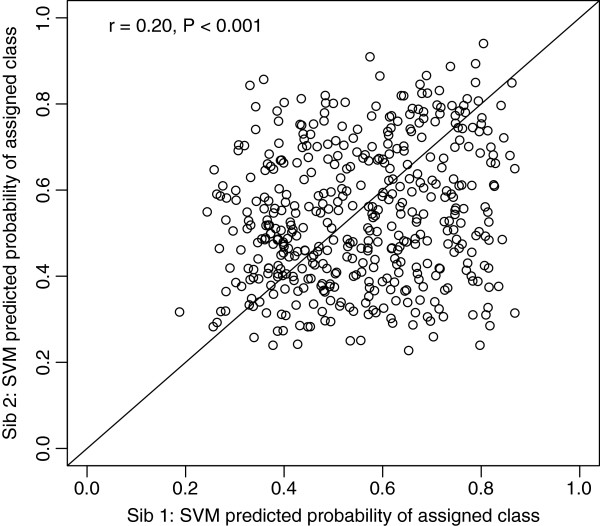
**Correlation of SVM predicted probabilities between AGRE siblings.** AGRE, Autism Genetics Resource Exchange; SVM, support vector machine.

Significant dependence between the class assignment of siblings in AGRE1 and the other sibling in AGRE2 was indicated (X-squared = 43, df = 25, *P* = 0.015). Furthermore, the predicted probabilities for the assigned class in AGRE1 (sib1) were significantly correlated with the predicted probabilities of their affected sibling AGRE2 (sib2) (Pearson’s correlation r = 0.20, *P* <0.001) (Figure 5). To exclude the possibility that these correlations were driven by severity rather than specificity of ADI-R profiles, we found that the severity of the proband symptom scores did not predict the predicted probability of its sibling, while the predicted probability scores did predict the probability score of the sibling (sibling 1 as predictor of sibling 2: mean items score *P* = 0.18; probability score *P* = 1.5e-05; sibling 2 as predictor of sibling 1: mean items score *P* = 0.86; probability score *P* = 7e-05).

Interestingly, the correlation in prediction probabilities was driven by a correlation (r = 0.35) between sib pairs assigned to the same class compared with ‘discordant’ sibs (r = -0.18), that is sibling pairs that had not been assigned to the same class. In addition, we found that the covariance in probabilities between sibs was greater when both sibs were assigned to the same genetic disorder class (F-test for equality of variances of the difference in probability, *P* <0.001). To confirm the notion of enhanced behavioral similarity between siblings allocated to the same genetic disorder class, we examined the ADI-R scores directly. We used the first principle component (PC1) of the ADI-R scores as a summary measure. Overall (disregarding genetic disorder class), the PC1s of sibs were not significantly correlated (r = 0.081, *P* = 0.089), but when split out for concordance of genetic disorder prediction, the correlations were 0.71 and -0.16 for concordant sibs and discordant sibs, respectively, with *P* <0.001 for ‘concordant’ versus ‘discordant’ sibs. Overall, the sibling analysis indicated that the familial liability to ASD may be partitioned according to the relative likelihood of disturbance related to certain genetic disorders.

## Discussion

This study demonstrates that patterns of autistic symptomatology can be associated with specific genetic disorders. There has been much speculation that such genotype-phenotype correlations exist but so far only limited evidence to support the conjecture. Our results are consistent with findings from animal research and suggest that different pathophysiological pathways underlie certain behavioral deficits [4,45].

The current study is the first to test the specificity of genetic behavioral phenotypes using a machine learning paradigm. The ADI-R algorithm items comprised a comparatively small number of symptom features, yet we used this small set of items to classify our cases. The total number of correct allocations (63%) was substantial given the fact that five groups were compared. Indeed, this result was derived from one-by-one genetic disorder comparisons, in which strong contrast were evident. It was notable, however, that the SVM algorithm derived from the current sample differentiated between some classes better than others. This variability might be explained by the variation in sample sizes; thus, in future larger samples will need to be investigated. It was also notable that the ratings of the pattern of social dysfunction were among the best contributors to class prediction, raising the possibility that particular styles of social impairment may be related to particular genetic risk factors. Although differences in the typology of social impairments have been noted in ASD [46], differences in the types of social impairment have not been studied in detail and are only partially captured by the ADI-R items. For instance, social avoidance is commonly reported in fragile X syndrome, as another example of social behavioral specificity within a genetic disorder associated with ASD [47,48]. It seems likely that with the incorporation of more symptoms and other phenotypic features, such as the presence of comorbid behavioral problems like those associated with ADHD [49], the ability to assign cases to specific classes of genetic disorder may be improved. The inclusion of other conditions such as fragile X syndrome may also help further map the patterns of genotype-phenotype correlations. Together, these extensions may reveal further contrasts or overlaps between genetic disorders that are biologically meaningful. For instance, it was already interesting that the prediction probabilities for SMC15 were similar to those for PWS. Both disorders are associated with abnormalities in the dosage of genes located in the 15q11-13 region and likely lead to perturbations in similar pathophysiological pathways.

The subjects of this study were included because they were ascertained for the presence of a genetic disorder and were assessed regardless of the presence or absence of behavioral concerns. Although this approach is likely to have minimized ascertainment biases, some bias cannot be ruled out. However, any enrichment of behavioral abnormalities in these cohorts is unlikely to give rise to the specific patterns of associations identified here. It was reassuring in this respect that the algorithm derived from all cases in the genetic disorder samples gave comparable results to the analyses that included only the subjects who scored above the ADI-R threshold for ASD. Analysis confirmed that IQ did not seem to act as a confounding factor in the SVM predictions. Also, the influence of age and medication as cofounds could be ruled out, as the ADI-R algorithm codes behaviors between 4 and 5 years old [35].

The application of the genetic disorder algorithm to AGRE samples indicated that the behavioral patterns observed in cases of idiopathic autism were not random. Therefore, these results could be used to estimate relative similarity to behavioral profiles designated from the genetic disorders. In addition, the sibling analysis showed correlation of SVM predictions between affected sib pairs. These findings indicate the feasibility to partition familiality into components according to patterns of autistic symptomatology, for example concordance in relative similarity to behavioral profiles related to the genetic disorders. This notion should be followed up by studies that incorporate genetic or pathway information to ascertain the behavior-based stratification in idiopathic samples. For instance, our allocation in idiopathic ASD to TSC-derived patterns may be supported by molecular data showing mammalian target of rapamycin (mTOR) pathway deregulation. Such a result would support the view that perturbation of the mTOR signaling cascade is a common pathophysiological feature of human neurological disorders, including mental retardation syndromes and ASDs [49]. If confirmed, such results could complement future gene searches, since stratification on the basis of behavioral profile may significantly increase the power to detect which (combination of) genetic disorder related pathways are most prominently involved. Indeed, the notion that pathophysiological processes are shared in syndromic and idiopathic cases of ASD is supported by a recent study that showed converging synaptic pathophysiology between syndromic (for example as a cause of a defined genetic disorder) and non-syndromic rodent models of autism [50]. Moreover, genotype stratification may also have important treatment implications, as other animal studies suggest that the best treatment approaches for some genetic disorders (for example fragile X syndrome) may be unsuitable for others (for example tuberous sclerosis) [49].

## Conclusion

Our proof of concept study indicates the existence of ‘signature’ autistic behavioral profiles that index underlying genetic risk processes. These signatures may be helpful in disentangling the etiological and phenotypic heterogeneity evident in ASD, but warrant replication in larger and independent samples. The approach presented in this study could hold promise as a means of stratifying patients who may benefit from treatments targeted at specific pathways and as a way of identifying those patients in whom interventions may have unwanted effects.

## Abbreviations

22q11DS: 22q11.2 deletion syndrome; ADI-R: Autism Diagnostic Interview-Revised; AGRE: Autism Genetics Resource Exchange; ASD: Autism spectrum disorder; CNV: Copy number variant; CREC: College Research Ethics Committee; DS: Down’s syndrome; IQ: Intelligence quotient; LOOCV: Leave-one-out cross-validation; METC: Medical Research Ethics Committee; mTOR: Mammalian target of rapamycin; PCA: Principal component analysis; PWS: Prader-Willi syndrome; SMC15: Supernumerary marker chromosome 15; SVM: Support vector machine; TSC: Tuberous sclerosis complex; XXY: Klinefelter syndrome.

## Competing interests

The authors declare no conflicts of interest.

## Authors’ contributions

HB designed the study, analyzed and interpreted the data, and drafted and revised the manuscript. ME designed the study, analyzed and interpreted the data, and drafted and revised the manuscript. MK undertook data interpretation, and drafted and revised the manuscript. SC analyzed and interpreted the data, and drafted and revised the manuscript. JV designed the study, analyzed and interpreted the data, and drafted and revised the manuscript. PB designed the study, analyzed and interpreted the data, and drafted and revised the manuscript. All authors read and approved the final manuscript.

## References

[B1] Betancur C Etiological heterogeneity in autism spectrum disorders: more than 100 genetic and genomic disorders and still counting Brain Res 2011 1380 42 77 21129364 10.1016/j.brainres.2010.11.078

[B2] Abrahams BS Geschwind DH Advances in autism genetics: on the threshold of a new neurobiology Nat Rev Genet 2008 9 5 341 355 10.1038/nrg2346 18414403 PMC2756414

[B3] Pinto D Pagnamenta AT Klei L Anney R Merico D Regan R Conroy J Magalhaes TR Correia C Abrahams BS Almeida J Bacchelli E Bader GD Bailey AJ Baird G Battaglia A Berney T Bolshakova N Bölte S Bolton PF Bourgeron T Brennan S Brian J Bryson SE Carson AR Casallo G Casey J Chung BH Cochrane L Corsello C Functional impact of global rare copy number variation in autism spectrum disorders Nature 2010 466 7304 368 372 10.1038/nature09146 20531469 PMC3021798

[B4] Sanders SJ Ercan-Sencicek AG Hus V Luo R Murtha MT Moreno-De-Luca D Chu SH Moreau MP Gupta AR Thomson SA Mason CE Bilguvar K Celestino-Soper PB Choi M Crawford EL Davis L Wright NR Dhodapkar RM DiCola M DiLullo NM Fernandez TV Fielding-Singh V Fishman DO Frahm S Garagaloyan R Goh GS Kammela S Klei L Lowe JK Lund SC Multiple recurrent de novo CNVs, including duplications of the 7q11.23 Williams syndrome region, are strongly associated with autism Neuron 2011 70 5 863 885 10.1016/j.neuron.2011.05.002 21658581 PMC3939065

[B5] Scherer SW Dawson G Risk factors for autism: translating genomic discoveries into diagnostics Hum Genet 2011 130 1 123 148 10.1007/s00439-011-1037-2 21701786

[B6] Cook EH Jr Scherer SW Copy-number variations associated with neuropsychiatric conditions Nature 2008 455 7215 919 923 10.1038/nature07458 18923514

[B7] Freitag CM The genetics of autistic disorders and its clinical relevance: a review of the literature Mol Psychiatry 2007 12 1 2 22 10.1038/sj.mp.4001896 17033636

[B8] Levy D Ronemus M Yamrom B Lee YH Leotta A Kendall J Marks S Lakshmi B Pai D Ye K Buja A Krieger A Yoon S Troge J Rodgers L Iossifov I Wigler M Rare de novo and transmitted copy-number variation in autistic spectrum disorders Neuron 2011 70 5 886 897 10.1016/j.neuron.2011.05.015 21658582

[B9] Toro R Konyukh M Delorme R Leblond C Chaste P Fauchereau F Coleman M Leboyer M Gillberg C Bourgeron T Key role for gene dosage and synaptic homeostasis in autism spectrum disorders Trends Genet 2010 26 8 363 372 10.1016/j.tig.2010.05.007 20609491

[B10] Liu XQ Paterson AD Szatmari P Genome-wide linkage analyses of quantitative and categorical autism subphenotypes Biol Psychiatry 2008 64 7 561 570 10.1016/j.biopsych.2008.05.023 18632090 PMC2670970

[B11] Fountain C Winter AS Bearman PS Six developmental trajectories characterize children with autism Pediatrics 2012 129 5 e1112 1120 10.1542/peds.2011-1601 22473372 PMC3340586

[B12] Bruining H de Sonneville L Swaab H de Jonge M Kas M van Engeland H Vorstman J Dissecting the clinical heterogeneity of autism spectrum disorders through defined genotypes PLoS One 2010 5 5 e10887 10.1371/journal.pone.0010887 20526357 PMC2878316

[B13] Hall SS Lightbody AA Hirt M Rezvani A Reiss AL Autism in fragile X syndrome: a category mistake? J Am Acad Child Adolesc Psychiatry 2010 49 9 921 933 10.1016/j.jaac.2010.07.001 20732628 PMC2943372

[B14] Smith LE Barker ET Seltzer MM Abbeduto L Greenberg JS Behavioral phenotype of fragile X syndrome in adolescence and adulthood Am J Intellect Dev Disabil 2012 117 1 1 17 10.1352/1944-7558-117.1.1 22264109 PMC3388941

[B15] Lincoln AJ Searcy YM Jones W Lord C Social interaction behaviors discriminate young children with autism and Williams syndrome J Am Acad Child Adolesc Psychiatry 2007 46 3 323 331 10.1097/chi.0b013e31802b9522 17314718

[B16] Pride NA Payne JM North KN The impact of ADHD on the cognitive and academic functioning of children with NF1 Dev Neuropsychol 2012 37 7 590 600 10.1080/87565641.2012.695831 23066937

[B17] Flores CG Valcante G Guter S Zaytoun A Wray E Bell L Jacob S Lewis MH Driscoll DJ Cook EH Jr Kim SJ Repetitive behavior profiles: consistency across autism spectrum disorder cohorts and divergence from Prader-Willi syndrome J Neurodev Disord 2011 3 4 316 324 10.1007/s11689-011-9094-3 21881965 PMC3261264

[B18] Oliver C Berg K Moss J Arron K Burbidge C Delineation of behavioral phenotypes in genetic syndromes: characteristics of autism spectrum disorder, affect and hyperactivity J Autism Dev Disord 2011 41 8 1019 1032 10.1007/s10803-010-1125-5 21080217

[B19] Moss J Howlin P Autism spectrum disorders in genetic syndromes: implications for diagnosis, intervention and understanding the wider autism spectrum disorder population J Intellect Disabil Res 2009 53 10 852 873 10.1111/j.1365-2788.2009.01197.x 19708861

[B20] Siegel MS Smith WE Psychiatric features in children with genetic syndromes: toward functional phenotypes Pediatr Clin North Am 2011 58 4 833 864 10.1016/j.pcl.2011.06.010 21855710

[B21] Li H Fertuzinhos S Mohns E Hnasko TS Verhage M Edwards R Sestan N Crair MC Laminar and columnar development of barrel cortex relies on thalamocortical neurotransmission Neuron 2013 79 5 970 986 10.1016/j.neuron.2013.06.043 24012009 PMC3768017

[B22] Bruining H Swaab H Kas M van Engeland H Psychiatric characteristics in a self-selected sample of boys with Klinefelter syndrome Pediatrics 2009 123 5 e865 870 10.1542/peds.2008-1954 19364768

[B23] Dennis NR Veltman MW Thompson R Craig E Bolton PF Thomas NS Clinical findings in 33 subjects with large supernumerary marker(15) chromosomes and 3 subjects with triplication of 15q11-q13 Am J Med Genet A 2006 140 5 434 441 16470730 10.1002/ajmg.a.31091

[B24] Milner KM Craig EE Thompson RJ Veltman MW Thomas NS Roberts S Bellamy M Curran SR Sporikou CM Bolton PF Prader-Willi syndrome: intellectual abilities and behavioural features by genetic subtype J Child Psychol Psychiatry 2005 46 10 1089 1096 10.1111/j.1469-7610.2005.01520.x 16178933

[B25] Vorstman JA Morcus ME Duijff SN Klaassen PW Heineman-de Boer JA Beemer FA Swaab H Kahn RS van Engeland H The 22q11.2 deletion in children: high rate of autistic disorders and early onset of psychotic symptoms J Am Acad Child Adolesc Psychiatry 2006 45 9 1104 1113 10.1097/01.chi.0000228131.56956.c1 16926618

[B26] Bolton PF Park RJ Higgins JN Griffiths PD Pickles A Neuro-epileptic determinants of autism spectrum disorders in tuberous sclerosis complex Brain 2002 125 Pt 6 1247 1255 12023313 10.1093/brain/awf124

[B27] Roach ES Gomez MR Northrup H Tuberous sclerosis complex consensus conference: revised clinical diagnostic criteria J Child Neurol 1998 13 12 624 628 10.1177/088307389801301206 9881533

[B28] Mullen EM Mullen Scales of Early Learning 1995 AGS American Guidance Service Inc: Circle Pines, MN

[B29] Raven JC Colored Progressive Matrices Sets I and II 1995 Oxford: Oxford Psychologists Press Ltd

[B30] Wechsler D Wechsler Preschool and Primary Scale of Intelligence-Revised 1989 New York, NY: Psychological Corporation

[B31] Wechsler D Wechsler Adult Intelligence Scale-Third Edition 1997 San Antonio, TX: Psychological Corporation

[B32] Snijders JT Tellegen PJ Winkel M Laros JA SON-R 2, 5–7 Niet-verbaleIntelligentietest-Revisie [SON-R 2, 5–7 Snijders-Oomen Non-verbal Intelligence Test-Revised] 2009 Lisse: Swets & Zeitlinger

[B33] Lajonchere CM Changing the landscape of autism research: the autism genetic resource exchange Neuron 2010 68 2 187 191 10.1016/j.neuron.2010.10.009 20955925 PMC3004528

[B34] Bucan M Abrahams BS Wang K Glessner JT Herman EI Sonnenblick LI Alvarez Retuerto AI Imielinski M Hadley D Bradfield JP Kim C Gidaya NB Lindquist I Hutman T Sigman M Kustanovich V Lajonchere CM Singleton A Kim J Wassink TH McMahon WM Owley T Sweeney JA Coon H Nurnberger JI Li M Cantor RM Minshew NJ Sutcliffe JS Cook EH Genome-wide analyses of exonic copy number variants in a family-based study point to novel autism susceptibility genes PLoS Genet 2009 5 6 e1000536 10.1371/journal.pgen.1000536 19557195 PMC2695001

[B35] Lord C Rutter M Le Couteur A Autism diagnostic interview-revised: a revised version of a diagnostic interview for caregivers of individuals with possible pervasive developmental disorders J Autism Dev Disord 1994 24 5 659 685 10.1007/BF02172145 7814313

[B36] Brune CW Kim SJ Salt J Leventhal BL Lord C Cook EH Jr 5-HTTLPR genotype-specific phenotype in children and adolescents with autism Am J Psychiatry 2006 163 12 2148 2156 10.1176/appi.ajp.163.12.2148 17151167

[B37] Kates WR Antshel KM Fremont WP Shprintzen RJ Strunge LA Burnette CP Higgins AM Comparing phenotypes in patients with idiopathic autism to patients with velocardiofacial syndrome (22q11 DS) with and without autism Am J Med Genet A 2007 143A 22 2642 2650 10.1002/ajmg.a.32012 17937445

[B38] Szatmari P Liu XQ Goldberg J Zwaigenbaum L Paterson AD Woodbury-Smith M Georgiades S Duku E Thompson A Sex differences in repetitive stereotyped behaviors in autism: implications for genetic liability Am J Med Genet B Neuropsychiatr Genet 2012 159B 1 5 12 10.1002/ajmg.b.31238 22095612

[B39] Liu XQ Georgiades S Duku E Thompson A Devlin B Cook EH Wijsman EM Paterson AD Szatmari P Identification of genetic loci underlying the phenotypic constructs of autism spectrum disorders J Am Acad Child Adolesc Psychiatry 2011 50 7 687 696 10.1016/j.jaac.2011.05.002 21703496 PMC3593812

[B40] Molloy CA Keddache M Martin LJ Evidence for linkage on 21q and 7q in a subset of autism characterized by developmental regression Mol Psychiatry 2005 10 8 741 746 10.1038/sj.mp.4001691 15940295

[B41] Flax JF Hare A Azaro MA Vieland VJ Brzustowicz LM Combined linkage and linkage disequilibrium analysis of a motor speech phenotype within families ascertained for autism risk loci J Neurodev Disord 2010 2 4 210 223 10.1007/s11689-010-9063-2 21125004 PMC2974936

[B42] Buitelaar JK Van der Gaag R Klin A Volkmar F Exploring the boundaries of pervasive developmental disorder not otherwise specified: analyses of data from the DSM-IV Autistic Disorder Field Trial J Autism Dev Disord 1999 29 1 33 43 10.1023/A:1025966532041 10097993

[B43] de Bildt A Sytema S Ketelaars C Kraijer D Mulder E Volkmar F Minderaa R Interrelationship between autism diagnostic observation schedule-generic (ADOS-G), autism diagnostic interview-revised (ADI-R), and the diagnostic and statistical manual of mental disorders (DSM-IV-TR) classification in children and adolescents with mental retardation J Autism Dev Disord 2004 34 2 129 137 15162932 10.1023/b:jadd.0000022604.22374.5f

[B44] R Development Core Team R: A Language and Environment for Statistical Computing 2005 Vienna: R Foundation for Statistical Computing

[B45] Kas MJ Fernandes C Schalkwyk LC Collier DA Genetics of behavioural domains across the neuropsychiatric spectrum; of mice and men Mol Psychiatry 2007 12 4 324 330 10.1038/sj.mp.4001979 17389901

[B46] Wing L Gould J Severe impairments of social interaction and associated abnormalities in children: epidemiology and classification J Autism Dev Disord 1979 9 1 11 29 10.1007/BF01531288 155684

[B47] Budimirovic DB Bukelis I Cox C Gray RM Tierney E Kaufmann WE Autism spectrum disorder in Fragile X syndrome: differential contribution of adaptive socialization and social withdrawal Am J Med Genet A 2006 140A 17 1814 1826 10.1002/ajmg.a.31405 16906564

[B48] Kau AS Tierney E Bukelis I Stump MH Kates WR Trescher WH Kaufmann WE Social behavior profile in young males with fragile X syndrome: characteristics and specificity Am J Med Genet A 2004 126A 1 9 17 10.1002/ajmg.a.20218 15039968

[B49] Auerbach BD Osterweil EK Bear MF Mutations causing syndromic autism define an axis of synaptic pathophysiology Nature 2011 480 7375 63 68 10.1038/nature10658 22113615 PMC3228874

[B50] Baudouin SJ Gaudias J Gerharz S Hatstatt L Zhou K Punnakkal P Tanaka KF Spooren W Hen R De Zeeuw CI Vogt K Scheiffele P Shared synaptic pathophysiology in syndromic and nonsyndromic rodent models of autism Science 2012 338 6103 128 132 10.1126/science.1224159 22983708

